# PROTOCOL: Is the CEO/employee pay ratio related to firm performance in publicly traded companies?

**DOI:** 10.1002/cl2.70003

**Published:** 2024-11-04

**Authors:** Denise M. Rousseau, Cédric Velghe, Ryan Splenda, Byeong Jo Kim, Jangbum Lee

**Affiliations:** ^1^ Heinz College and Tepper School of Business Carnegie Mellon University Pittsburgh Pennsylvania USA; ^2^ The VIGOR Unit Ghent Belgium; ^3^ Carnegie Mellon University, University Libraries Pittsburgh Pennsylvania USA; ^4^ Graduate School of Public Administration Seoul National University Seoul Korea

**Keywords:** CEO compensation, CEO/employee pay ratio, employee compensation, firm performance

## Abstract

One goal of this systematic review is to assess whether the pay ratio, that is, the relative difference between the compensation a firm's CEO receives and that of its nonmanagerial employees, is related to subsequent firm performance. A second goal is to identify factors influencing this relationship across publicly traded firms, including the pay ratio's perceived fairness by employees, the firm's business strategy, and related factors.

## 1 BACKGROUND

### 1.1 The problem, condition, or issue

Pay ratio refers to the relative difference between CEO compensation and employee compensation (Alan et al., 2021; Faleye et al., 2013; Rouen, 2020), the latter typically assessed as the median of nonmanagerial employee compensation. Attention to the pay ratio by policy makers and the public is motivated by the rise in CEO pay over the past 40+ years. From 1978 to 2022, CEO compensation shot up 1,209.2% compared with a 15.3% increase in a typical worker's compensation with CEOs paid 344 times as much as the typical worker (Bivans & Kandra, 2023). The value of high CEO compensation to attract capable leadership and enhance business outcomes is highlighted in economic research (Falato et al., 2015; Gan & Park, 2016) while economists have also questioned whether high CEO rewards are bestowed with too little consideration for their actual effect on to firm performance (Bebchuk & Fried, 2009). This systematic review examines the relationship of the CEO/Employee Pay Ratio with firm performance in research conducted in the time frame since reports of CEO/Employee Pay Ratio were mandated. It seeks to inform both company board/compensation committees and HR leaders regarding the implications of pay practices for the firm.

### 1.2 The intervention

In general, pay ratio refers to the relative difference in compensation between senior executives and a firm's workers. Typically research on pay ratio compares CEOs to workers (Alan et al., 2021; Faleye et al., 2013; Newton, 2015; Rouen, 2020) or to the top management team (i.e., “pay slice,” Henderson & Fredrickson, 2001; Siegel & Hambrick, 2005); it also compares compensation for the top management team relative to employees (Bamberger et al., 2021; Cowherd & Levine, 1992). The focus of this review is the CEO/worker pay ratio, that is, the relative difference between CEO compensation and employee compensation (Alan et al., 2021; Faleye et al., 2013; Roen, 2015). Considerable contention exists regarding the pay‐outs CEOs receive, whether poor firm performance actually limits CEO compensation (Bebchuk & Fried, 2009), and whether financial incentives have any consistent effect on firm performance (Rousseau et al., 2023). This review focuses on the effects of the annual pay ratio derived from public reports of CEO compensation and the median compensation received by employees in the same year.

### 1.3 How the intervention might work

Scholars espouse two theories to account for the relationship between the pay ratio and firm outcomes. Economic theory posits a positive effect on firm performance as the result of a motivating incentive contract allowing a tournament, where the higher performer is paid higher than others to whom they compare themselves (Eriksson, 1999). A finding of a positive relationship is interpreted as appropriately aligned incentives (Jensen & Murphy, 1990). Fairness theory argues for a negative relationship between pay ratio and firm performance based on perceptions of unfair or inequitable pay (Colquitt & Zipay, 2015). A finding of a negative relationship between pay ratio and product quality has been interpreted as employees’ withholding their contributions to the firm due to perceived unfairness (cf Cowherd & Levine, 1992).

Distinct mechanisms underlie theories predicting positive or negative effects of pay ratios on firm performance. For the positive effect posited by agency theory, two conditions are required. First, the CEO's ability and motivation to enhance firm performance would need to be amplified by being highly paid. Ability is enhanced if more qualified and capable CEOs are paid more than their less qualified counterparts leading to greater attraction and retention of more highly qualified CEOs (De Angelis & Grinstein, 2020; Falato et al., 2015; Uygur, 2019). Motivation is enhanced if high pay increases CEO efforts on the firm's behalf while lower pay reduces those efforts. Second, the factors influencing firm performance need to some extent to be under the chief executive's control (e.g., innovation rates that increase competitive position) rather than due to market factors alone (Bertrand & Mullainathan, 2001). For the negative effect posited by fairness theory, workers would need to know the general level of CEO compensation to form fairness judgments, and thus seek to restore equity by withholding contributions (cf Cowherd & Levine, 1992). Table 1

**Table 1:** Campbell standards for conduct and reporting, 2024.

Section of review	Check if done
**1. Scope of the review** Review questions need to be clear and address the issues important to stakeholders. Use an appropriate framework that captures the elements of your question (e.g., PICO for intervention reviews) to develop the intervention review question and objectives while particularly defining the outcomes of interest. For non‐intervention reviews, similarly develop the comparable elements, such as the population and critical variables (e.g., risk factors and outcomes). Ensuring that the questions address issues that are important to consumers, practitioners, policymakers, scholars, and others. If the review will address multiple interventions, clarify how these will be addressed (e.g., summarized separately, combined, or explicitly compared). If applicable, issues related to populations experiencing inequities and adverse effects should be considered as part of the review questions and objectives. Heterogeneity could impact the scope of the review. If heterogeneity is expected, the likely sources of heterogeneity should be defined, and subsequently, moderator analyses should be clearly described. Deviations from the original questions should be justified and reported accordingly.	X
a) Formulate clear review questions	X
b) Indicate relevance to policy and decision‐makers needs	X
c) Consider issues related to populations experiencing inequities and how these will be considered	X (workers)
d) Discuss issues related to adverse effects and how these will be assessed	NA
e) Pre‐define clear objectives	X
**2. Eligibility criteria** Pre‐defined, unambiguous eligibility criteria are a fundamental prerequisite for a systematic review. Thus, it is important to construct eligibility criteria that align with the PICO (or alternative relevant framework for non‐intervention reviews) in the review questions and the objectives that other research teams can replicate. These eligibility criteria should be clear and well justified. The population could be rationally specified by setting, age, identifying personal characteristics, demographic factors, and other factors that differentiate the participants. Specification of comparator interventions requires particular clarity, including the extent to which the interventions are compared with a control or comparison conditions with matched or similar participants. Outcome measures are not necessarily part of the eligibility criteria of a review. However, some reviews do legitimately restrict eligibility to specific outcomes. For example, the same intervention may be studied in the same population for different purposes (e.g., reading interventions); or a review may address specifically the adverse effects of an intervention used for several conditions. In such cases, the outcomes for this review should be explicitly mentioned. Study designs should be defined with their features rather than labels and selected based on their appropriateness for the review. Studies should not be excluded on the basis of availability of outcome data since this may introduce outcome reporting bias. Studies with no available data but which meet eligibility criteria should be listed as included studies. If the review will exclude studies due to the risk of potential bias, there should be rationale for this decision as well as detailed information provided about how the review authors will determine and document the risk of bias during the eligibility review process.	
a. Describe population	X
b. Describe intervention (or predictor)	X
c. Describe comparator	Continuous predictor variable
d. Pre‐specify primary and secondary outcomes	X
e. Pre‐define study designs	X Cross‐section and longitudinal will be identified and compared)
f. Pre‐define additional study factors (e.g., Time frame/geographical scope)	X
**3. Search strategy** The goal of the search is to identify all eligible studies and to do so in a way that is transparent, replicable, and reduces the potential to further publication bias. To do this one needs to search multiple databases as well as gray literature and the reference lists of existing reviews and included studies. Additional methods, such as conducting a forward citation search of seminal works and included studies and hand searching key journals, can be used to improve comprehensiveness. The searches of databases should involve a well‐thought‐out set of keywords and make effective use of Boolean logic, wild cards, phrase searching, and subject headings, although one should be cautious of using some built‐in database filters. If possible, involve a librarian or information retrieval specialist with systematic review experience. A careful log of the search should be maintained and reported in the final review, including when the search was conducted, the results of the search, database and platform names, and the exact search strategy for each database. This includes all keywords, subject headings, and database syntax used for each bibliographic database and for gray literature resources when applicable. It is important to search for unpublished studies to mitigate the influence of reporting bias, including contacting experts in the field.	
a. Describe search strategy in enough detail for replication (as described above)	X
b. Develop a search strategy that has a reasonable probability of identifying all studies that match eligibility criteria	X
**4. Screening and Inclusion** The goal of screening studies for inclusion is to ensure that inclusion/exclusion decisions are transparent and reproducible. Screening is typically done in two stages. The first is a screening of the titles and abstracts obtained from the search strategy and the goal is to identify those titles and abstracts that are potentially eligible and should be moved to the second stage. At the second stage, studies are screened for inclusion against the eligibility criteria. Throughout, the study team should maintain sufficient documentation for the creation of a PRISMA flowchart. Screening in duplicate will help reduce screening errors—i.e., missing studies that should be included or erroneously including ineligible studies. The report should include a table of included and studies excluded at the full‐text screening (second stage). The list of excluded studies should list those studies which appear to meet inclusion criteria, i.e., “near misses” and provide justification for exclusion. If automation is used, describe how, including any validation, if used.	
a. Describe screening process and how it is designed to maximize reliability and replicability	X
b. Document the sources and flow of studies, with reasons for exclusion, using a PRISMA flowchart	NO SEARCH YET
**5. Coding and data extraction** The goal of the coding process is to extract descriptive and statistical information in a fashion that is reliable and could be replicated by other researchers. The coding protocol should include both descriptive and statistical information needed for calculating effect sizes and produce needed tables (i.e., a table of study characteristics, risk‐of‐bias, etc.). Descriptive details should include details of the intervention, population, methods of outcome assessment, setting, etc. that would allow replication, as well as factors that might be associated with risks of bias such as funding, whether the study is led by the program developer, or other factors. Information to inform critical appraisal is essential to collect as it is an integral component of high caliber evidence syntheses. Critical appraisal should be carried out with an appropriate risk of bias tool that includes assessment of domains such as selection bias, confounding, attrition that are appropriate for the study designs that are included. Additional information may be needed for planned or post hoc analyses, such as meta‐regression. Study coding should make use of a coding protocol with clearly defined variables and guidance on how to handle borderline cases (i.e., difficult coding decisions). When coding is conducted in duplicate, differences could be resolved either through consensus or by a third coder. When multiple manuscripts are available for a single study, use all available information for making coding decisions. It is best practice to reach out to authors for missing critical information about the study (e.g., such as information needed to calculate an effect size), and these efforts to contact authors should be documented, and ideally reach out to all authors.	
a. Describe data extraction process (e.g., who is involved, use of pre‐tested forms, machine learning, etc.) and how these are designed to minimize bias	X
b. Specify and provide rationale for each effect size index	X CURVILINEAR AND LINEAR
c. Use all available manuscripts from the same study	X
d. Seek key information that is missing	X
e. Conduct critical appraisal assessment (e.g., assessment of risk of bias or study quality fit for the purpose of the review)	X
**6. Synthesis methods** The synthesis methods should be well justified and use widely accepted meta‐analytic methods or in the case of a qualitative review, an appropriate and established approach. Plan in advance the methods to be used to synthesize the results of the included studies, including whether a quantitative synthesis is planned, how heterogeneity will be assessed, choice of effect measure (e.g., standardized mean difference, odds ratio, risk ratio, correlation, incident rate ratio, among others), how you will handle multiple effect sizes within studies and methods for meta‐analysis (e.g., fixed‐effect or random‐effects model). There are several estimators of the random effects variance component. The restricted maximum likelihood (REML) estimator is considered a good default but others are better suited to particular analytic situations. Heterogeneity affects the extent to which generalizable conclusions can be formed. It is important to identify heterogeneity in case there is sufficient information to explain it and offer new insights. Heterogeneity should be reported using appropriate metrics (e.g., Tau2, *I* ^2^, etc.). When quantitative effectiveness data has been collected, vote counting is never an accepted method of synthesis. If a quantitative synthesis is not planned, or if it is not possible, plan the specific methods to narratively synthesize the results of the included studies. Regardless of the synthesis approach chosen, justification must be provided for the choice, including addressing why the selected model/estimation method is appropriate for the analysis. Visualizations of the data, including forest plots should be used to present findings. There is overwhelming evidence of reporting biases of various types. Analyses of the results of included studies, for example using funnel plots or regression tests for funnel plot asymmetry, can sometimes help determine the possible extent of the problem, as can attempts to identify study protocols, which should be a more routine feature of a review.	
a. Justify the choice of effect measure(s)	X
b. Justify choice of model and estimation method	X
c. Assess and report effect sizes, measures of variance, and heterogeneity	PLANNED
d. Use an appropriate method for handling dependencies among effect sizes	PLANNED
e. Examine heterogeneity if possible	PLANNED
f. Incorporate critical appraisal domains into moderator analysis, if possible	PLANNED
g. Explore publication and outcome selection bias	PLANNED
**7. Discussion** The interpretation of results should focus on the pattern of evidence across studies and do so in light of (1) the quality of the evidence (i.e., critical appraisal), (2) the heterogeneity or variability in results across studies, and (3) the directness of the evidence to the review question. The directness of the evidence refers to the degree to which the intervention, context, population and settings align with the research question. Interpretation should discuss the meaningfulness of the size of effect in relation to the population and the population being studied. For example, an effect size for education is often benchmarked against the expected increase in years of learning. Interpretation should also consider certainty which includes the concepts of precision, inconsistency, risk of bias, including publication selection bias, and directness of the evidence. Interpretation should not be based on the statistical significance of the pooled effect. Review authors should discuss what new studies are needed to address weaknesses in the evidence base and what new directions in research we need to pursue to improve our understanding of the topic. Policy and practice recommendations are desirable but be cautious to not go beyond the evidence identified in the review.	
a. Interpret magnitude of effects in light of the credibility of the evidence (e.g., critical appraisal of evidence; publication/reporting bias)	PLANNED
b. Discuss uncertainty around key findings (i.e., heterogeneity of findings)	PLANNED
c. Interpret findings in light of the directness of the evidence (generalizability/external validity/construct validity)	PLANNED
d. Identify key implications for practice and research, to the extent justified from 7a, 7b, 7c	PLANNED

The structure of our review and search is informed by these alternative mechanisms.

### 1.4 Why it is important to do this review

**Table:** 

No current review exists on this topic. In the United States, the Dodd‐Frank Wall Street Reform and Consumer Protection Act (Security and Exchange Commission, 2015) mandated that beginning in 2018 publicly traded companies report the “pay ratio” of the CEO to the median employee's pay. In the UK, such regulations were enforced beginning in 2020 for firms over 250 employees (“New executive pay transparency measures come into force”, 2019). However, at present, the EU has no such policy, although it is debated. No regulatory policy exists on the nature of the ratio. Absent a review on the effect of pay ratio on firm and employee outcomes, policy makers lack evidence to inform future pay‐ratio‐related decisions. This review seeks to evaluate the evidence for the potential effects of pay ratios on firm performance and employee outcomes, given that with transparency, workers, customers, policy makers, and the public are able to act based on this information.

## 2 OBJECTIVES

One goal of this systematic review is to assess whether pay ratio, that is, the relative difference between the compensation a firm's CEO receives and that of its nonmanagerial employees, is related to subsequent firm performance. A second goal is to identify factors influencing this relationship across publicly traded firms, including the pay ratio's perceived fairness by employees, the firm's business strategy, and related factors.

**Methods**

MECCIR Updated standards attached at end of this protocol.

### 2.1 Criteria for considering studies for this review

P = Publicly Traded Firms.
I = Reported CEO/Employee Pay Ratio since legally mandated (e.g., 2018 in US).
C = Continuous effects of “I” will be examined.
O = Firm Performance (Metrics include financial and market‐related indicators); Employee Satisfaction, Fairness Perceptions.

#### 2.1.1 Types of studies

*Study data from 2018 on*. This review focuses on studies using data from 2018 to the present, the US start of required reporting of CEO‐employee pay ratio. To provide an appropriate test for fairness as a mechanism underlying pay ratio effects, employees need access to information regarding their CEOs pay. In the United States, the Dodd–Frank Wall Street Reform and Consumer Protection Act in 2018 began requiring publicly traded companies to report this pay ratio while the UK introduced a similar requirement in 2020 for all companies with greater than 250 employees.

*Publicly traded firms*. Since access to financial outcomes and/or employee attitudinal data are required to address our question, this review focuses on publicly traded firms required to report both pay ratios and annual financial results.

*Study designs*. Organization‐level studies including both CEO/employee pay ratio data and either/or both firm performance and employee attitudinal data. Studies may be cross‐sectional or longitudinal. Inclusion requires that pay ratio is assessed before or concurrently with measures of firm performance and employee attitudes. Meta‐regression will test for effects of study design.

#### 2.1.2 Types of participants

Publicly traded companies for which pay ratio is required reporting.

#### 2.1.3 Types of interventions

N/A

#### 2.1.4 Types of outcome measures

*Primary outcomes*

Firm performance metrics including annual Return on Assets, Profitability, and Market outcomes (e.g., Tobin's *Q*).

*Secondary outcomes*

Employee attitudes including job satisfaction, outcome fairness, pay fairness, and related indicators.

*Duration of follow‐up*

N/A

*Types of settings*

Publicly traded organizations where financial reporting is made public.

### 2.2 Search methods for identification of studies

To address the question on the relation of CEO/Employee pay ratio and publicly traded firm's performance, we developed a search strategy that will help focus our search of the overall pay/compensation literature. Our search strategy aims to limit results to the following:

- Research reported since January 1, 2018 (this time frame is used to match when the US began requiring public companies to disclose “pay ratios” between CEOs and employees). The UK began to require this as of 2020. We will include research from any country after January 1, 2018 that meets our PICO but code whether the country had required reporting at the time of the data collection.
- Research focused on Chief Executive Officers/Executive Directors compensation.
- Research that measures publicly traded firm performance (both financial and market‐related).

The search strategy will be executed in a number of electronic search resources yielding a comprehensive corpus of research. These resources will include subject‐specific and interdisciplinary bibliographic literature databases and subject‐specific gray literature websites and repositories. We will not exclude potential studies based on publication states (e.g., preprints and working papers).

In the bibliographic literature databases, we will use a combination of thesaurus/subject terms and keywords to find relevant literature on the topics of interest related to the research question. Searches will be limited to specific metadata fields including titles, abstracts, and when available, author‐supplied keywords. For the gray literature websites and repositories, advanced keyword searching will be used if and when available. The subject/thesaurus terms and keywords will address CEOs and workers, pay ratio, and firm performance.

A hand search of relevant journals in which studies on this topic tend to be published, but that are not indexed in the bibliographic databases, will be performed to identify additional primary studies.

Additional relevant studies will be harvested from the references of the studies identified for inclusion, as well as related literature reviews. Titles of included studies will be searched in Google Scholar and references citing those titles will be reviewed for inclusion.

#### 2.2.1 Electronic searches

The primary resources used for searching and gathering relevant studies are subject‐specific and interdisciplinary bibliographic literature databases. The databases that will be used in our search are as follows:

- ABI/INFORM Collection (ProQuest).
- Business Source Premier (EBSCOhost).
- Web of Science Core Collection (Clarivate).
- KCI Korean Journal Database (Clarivate).
- Scopus (Elsevier).
- Dissertations and Theses Global (ProQuest).
- Directory of Open Access Journals (DOAJ): https://doaj.org/.

A search strategy developed for the ABI/INFORM database is included in the Supporting Information (Appendices) section of this protocol. This search will be translated across the above mentioned database platforms. Searches will be limited to studies reported from January 1, 2018, onward. Additionally, in ABI/INFORM and Business Source Premier, we will use available filters to remove news articles, trade journals, industry reports, magazine articles, and other non‐research outputs.

#### 2.2.2 Searching other resources

In addition to the bibliographic literature databases, we will search a number of gray literature resources that include conference proceedings and papers that are not indexed in electronic databases, working papers, white papers, and other types of information from January 1, 2018, onward. Basic keyword searches with terms related to CEOs, pay ratio, and firm performance will be performed in the following websites:

- National Bureau of Economic Research (NBER) Working Papers: https://www.nber.org/papers.
- Social Science Research Network (SSRN) via Elsevier: https://papers.ssrn.com/.
- The Conference Board—Business Management Research: https://www.conference-board.org/us/.
- Bureau of Economic Analysis (BEA) Papers: https://www.bea.gov/research/papers.
- Centre for Economic Policy Research (CEPR): https://cepr.org/.
- Board of Governors of the Federal Reserve System publications: https://www.fedsearch.org/board_public/.
- Google Scholar: https://scholar.google.com.

When advanced or structured searching mechanisms exist within these websites, we will include detailed search strategies for those instances. Any gray literature reported before January 1, 2018 will be excluded and a date limit will be applied to the search when possible. There will be one case where we apply more stringent date limiters to a gray literature source (NBER Working Papers). We will only search for literature from 1‐1‐2021, onward in NBER because the previous years are indexed in the ABI/INFORM database and relevant studies will be identified in the ABI/INFORM search strategy.

Another popular and commonly used gray literature repository of business, management, and economics working papers is the Research Papers in Economics (RePEc) IDEAS database/website. We will not search this site because its content is indexed in the ABI/INFORM database from 2000‐present. Since the parameters of our study include only research from January 1, 2018, onward, there is no need to search IDEAS since relevant studies will be identified in the ABI/INFORM search strategy.

Two of the above‐mentioned sites do not support basic or advanced searching techniques (The Conference Board and Centre for Economic Policy Research). Because of this, we will employ a keyword search that limits results to these two sites. For example:

- CEO AND “pay ratio” AND “firm performance” site:.conference-board.org.
- CEO AND “pay ratio” AND “firm performance” site:. cepr. org.

Due to the limitations in place for Google Scholar searching (e.g., 256 total character searching limit), we will use the same search strategy described for The Conference Board and CEPR sites (e.g., ceo AND “pay ratio” AND “firm performance”). Additionally, we will screen the first 100 Google Scholar results for relevant studies.

*Hand Searching*

Some hand searching will supplement the electronic and gray literature searching. We will screen tables of contents and reference sections in the following journals for additional relevant studies:

- *Advances in Business Research*: https://journals.sfu.ca/abr/index.php/abr.
- *Academy of Management Review* (past 1 year).
- *Academy of Management Annals* (past 1 year).

A number of business and management conferences produce conference proceedings reports or publications. Many of these are indexed in the abovementioned electronic bibliographic databases (e.g., The Academy of Management Proceedings in Business Source Premier). The following conference proceedings/publications are not indexed in databases and will be screened for relevant studies on their openly available websites starting from January 1, 2018:

- International Conference on Economics, Business and Management (ICEBM) ‐ Journal of Economics, Business and Management: https://www.joebm.com/list-6-1.html.
- International Conference on Advances in Management Sciences (ICAMS) ‐ Journal of Advanced Management Science: https://www.joams.com/index.php?m=content&c=index&a=lists&catid=9.
- Academy of International Business (AIB) Proceedings: https://www.aib.world/about/history/past-conferences/past-annual-meetings/.

References of included studies will be screened, as well as those in related literature reviewed published after January 1, 2018. Titles of included studies will also be searched in Google Scholar and references citing those titles will be reviewed for inclusion. Lastly, we will contact selected subject matter experts to determine whether there are additional in press or unpublished studies relevant to our systematic review. Subject matter experts can be determined on the results of the search strategies that have been outlined above in this section, and based on already established peer networks.

### 2.3 Data collection and analysis

#### 2.3.1 Description of methods used in primary research

Anticipated methods include company surveys where archival data on pay ratio and financial results are coupled with survey data on employee attitudes.

#### 2.3.2 Selection of studies

Using Covidence, two coders will independently screen studies to identify those meeting inclusion criteria. These coders will compare their decisions and any differences will be resolved through discussion.

#### 2.3.3 Data extraction and management

Two coders will separately extract data from each included study entering information into an excel file. Coding categories include study identifiers (bibliographic reference) and characteristics (population, method, data sources), key variables measured (I,O), control variables used, and study background information (time frame, country, etc.).

We will identify the most comprehensive model from each study, incorporating all reported covariates, and use the coefficients from these full models to calculate effect sizes. In cases where multiple eligible results are presented within a single study, our research team will conduct a thorough review and reach a consensus on the most representative effect size for that study. This approach ensures that we extract only one effect size per study, mitigating potential issues of dependency in our meta‐analysis. By consistently selecting the most comprehensive models and making collective decisions on representative effect sizes, we aim to enhance the robustness and comparability of our analysis while maintaining transparency in our methodology.

#### 2.3.4 Assessment of risk of bias in included studies

Our plan is to assess risk of bias in terms of the (1) nature and justification for control variables, (2) clarity of treatment for missing data and suitability of remedies applied, and (3) adequacy of tests for spurious effects or alternative explanations. We will finalize our framework for assessing risk of bias once we have identified the studies from which data will be extracted.

#### 2.3.5 Measures of treatment effect

“r” operationalized as partial regression coefficients. If a study reports both adjusted and unadjusted effects, we will extract both. For adjusted effects, we will register which covariates were included in the model, as well as their effect on the dependent variable.

Meta‐analysis combines samples from multiple studies to reach comprehensive conclusions, with effect sizes relative to sample sizes (Ringquist, 2013). We prioritize partial correlation coefficients to reflect relationships with other variables, avoiding bias from simple correlations (Fernández‐Castilla et al., 2019). To account for varying sample sizes, we convert correlation coefficients to Fisher's *Z* for effect size estimation. When relationships are statistically insignificant, we consider the effect size as 0 (Ringquist, 2013). Our effect size calculation prioritizes: first, calculating partial correlations from unstandardized regression coefficients (*B*), *t*‐values (standard errors), and degrees of freedom; second, using standardized regression coefficients (*β*) if provided; and third, utilizing simple correlation coefficients from correlation tables if regression results are unavailable. We calculate effect size variance as 1/(*n* − 3) and standard error as the square root of this variance. This approach ensures a comprehensive and statistically sound analysis of the relationship between CEO pay and firm performance across diverse studies.

#### 2.3.6 Unit of analysis issues

All included studies will report effects at the organizational level.

#### 2.3.7 Criteria for determination of independent findings

We will evaluate risk of nonindependence once we identify the included studies and observe their research methods and choice of sample. Multiple reports from a single study will be reviewed during extraction. How we will cope with multiplicity in effect sizes (i.e., multiple conceptually similar outcomes in a single study), depends on factors that we will only be able to oversee once we have collected all the eligible studies, for example, are these effect sizes interchangeable or equivalent (López‐López et al., 2018).

#### 2.3.8 Dealing with missing data

Most data to be coded will have been attained from public records and thus we anticipate few issues with missing data.

#### 2.3.9 Assessment of heterogeneity

We will employ partial correlation coefficients (partial r) as our primary effect size measure. In cases where partial r is not directly reported, we will derive it from regression coefficients. To address the variability in covariates across studies, we will extract partial r or regression coefficients from the most comprehensive model that includes all reported covariates. For studies featuring moderation terms, we will utilize the model excluding these terms to maintain consistency in effect size calculation.

For each aggregated effect size, we will report Q and perform the *χ* ^2^ test for heterogeneity. Since this test might be underpowered by a small number of studies, we will also report and interpret *T*² and *I*². Next, to assess the amount of variation in effect sizes across studies and its implications, we will also calculate and report the prediction interval. However, if the number of studies is small (i.e., <10), we will be particularly careful in interpreting the prediction interval (Borenstein et al., 2021).

#### 2.3.10 Assessment of reporting biases

We will include studies from gray literature and test for differences in effect sizes between published and unpublished studies, using meta‐regression techniques. We will also generate a funnel plot and visually inspect its symmetry. If sufficient studies (i.e., >10), we will also statistically test the funnel plot asymmetry. In case of funnel plot asymmetry, we will consider to which degree sources other than non‐reporting bias may explain this asymmetry (e.g., methodological quality, true heterogeneity).

To address potential publication bias, we will employ a comprehensive set of statistical methods, expanding beyond our initially proposed approach. In addition to the Peters regression test and funnel plot analysis mentioned in our sensitivity analysis, we will implement Egger's test and Begg's test, as originally planned, to statistically assess potential publication bias.

#### 2.3.11 Data synthesis

Since theories make competing predictions regarding the relationship of pay ratio to firm performance, we will test both linear and curvilinear effects. If authors fail to report sufficient information for both tests, we intend to contact them (which should be possible given the recent nature of relevant research).

We will use STATA to conduct a random‐effects meta‐analysis, using the “partial r” metric for our effect size indicator. Whereas fixed‐effect models assume that all studies reflect the same population, attributing any differences to sampling error, random‐effect models assume underlying effects follow a normal distribution. We will use inverse variance methods to weight study effect sizes by their precision in our meta‐analysis. We also anticipate that it might be more appropriate to synthesize the bivariate and partial effect sizes in separate meta‐analyses and only combine adjusted effects from studies that are based on the same regression model. Yet, we might consider approaches for combining adjusted effects from studies that are based on different regression models (Fernández‐Castilla et al., 2019), if appropriate.

To test for the curvilinear relationship between CEO/employee pay ratio and subsequent firm performance, we will follow the guide for conducting a curvilinear meta‐analysis of Mackey et al. (2020) and use a curvilinear (i.e., quadratic) term for our independent variable. If any of the studies do not provide results that include curvilinear terms, we will contact the authors to request additional information about their research. Two independent coders will enter the curvilinear correlation information into our data extraction form. Based on this data we will generate meta‐analytic correlation matrices. In line with Mackey et al. (2019), we will then run a meta‐regression, relative weight analysis, and a semipartial correlation procedure, which allows for assessing the relative contribution of the linear and curvilinear effects. The regression results examine the incremental contribution of the curvilinear terms while controlling for the effect of the linear terms. The relative weight analysis presents the amount of variance that the linear and curvilinear terms each predicted in the outcome. Adding the semi‐partial analysis permits assessing the consistency and robustness of the results across meta‐analytical techniques and identifying the unique variance (i.e., incremental validity) that is explained by the curvilinear term.

We acknowledge the challenge of ensuring complete uniqueness of firm data across studies. To address this concern, we will implement a multi‐faceted approach. First, we will create a comprehensive database of all included studies, detailing data sources, time periods covered, sample sizes, and key variables with their definitions. This will enable us to conduct a thorough cross‐comparison of studies to identify potential overlaps in data sources and time periods. In cases of significant overlap, we will prioritize studies with larger sample sizes or longer time periods, or consider including both studies but weight them appropriately in our analysis. To ensure full transparency, we will include this detailed study information in our meta‐analysis, allowing readers to assess potential data similarities. Additionally, we will conduct sensitivity analyses to evaluate the impact of potentially overlapping samples on our results. While this approach may not eliminate all instances of firm duplication, it will significantly mitigate potential biases and ensure transparency in our methodology, thereby enhancing the robustness and reliability of our findings.

#### 2.3.12 Subgroup analysis and investigation of heterogeneity

If the number of studies is sufficient we will test whether differences exist between countries and examine whether the set(s) of control variables (covariates) affect reported effect sizes.

As indicated in the background section of this protocol, we also plan to test whether the relationship between the CEO/employee pay ratio and subsequent firm performance is influenced by other factors (e.g., country, human resource practices or perceived fairness). If the number of studies is sufficient, we will test whether differences exist depending on the country in which the organizations are headquartered and on employee perceptions. Since it is a categorical variable, we will use the *Q*‐test to test whether the effect size varies according to the country. We will apply a random‐effects model as we expect that the heterogeneity is also attributable to other study differences (Borenstein et al., 2021). For employee perceptions as a continuous variable, we will perform a meta‐regression applying a random effects model to test if it is associated with the effect sizes in the studies. Following the same methods, we will, if possible, also examine whether heterogeneity can be explained by other study characteristics, including what firm performance metric was used, study design (i.e., cross‐sectional vs. longitudinal), firm strategy, human resource practices, the type of effect size (i.e., bivariate vs. partial), what controls or covariates were included in the analysis, and other factors related to our risk of bias judgments.

We appreciate the reviewer's suggestions regarding country‐based subgroup analysis. We will expand our approach by generating dummy variables for the US and UK, with other countries as the reference group. This method will allow for a three‐way comparison (US, UK, and other countries), providing a more nuanced understanding of country‐specific effects on the CEO pay‐firm performance relationship. Regarding the suggestion to incorporate power distance as a cultural value moderator using Hofstede's model, while we acknowledge its potential value, we regrettably lack comprehensive data on this dimension across our sample. We will note this as a limitation and suggest it as an avenue for future research.

#### 2.3.13 Sensitivity analysis

If possible given the information reported, we will test for effects of the methodology used to compute the pay ratio.

#### 2.3.14 Treatment of qualitative research

We do not plan to include qualitative research in this review.

#### 2.3.15 Summary of findings and assessment of the certainty of the evidence

We do not plan to include Summary findings and assessment of the certainty of the evidence.

## CONTRIBUTIONS OF AUTHORS

- Content: Denise M. Rousseau and Cédric Velghe, both are organizational psychologists with substantial research and consulting experience in areas of compensation and workplace practices.
- Systematic review methods: Denise M. Rousseau & Cédric Velghe, both have conducted a previous systematic review.
- Statistical analysis: Denise M. Rousseau, Cédric Velghe, Byeong Jo Kim, & Jangbun Lee, all have statistical expertise related to systematic reviews.
- Information retrieval: Ryan Splenda, is a business librarian and co‐author of a previous systematic review.

## DECLARATIONS OF INTEREST

No author has a conflict of interest related to the intervention or prior reviews.

## PRELIMINARY TIMEFRAME

We anticipate screening included studies as soon as the protocol is approved. Data extraction should be completed by end of September 2024 such that analyses can be conducted in Fall 2024.

## PLANS FOR UPDATING THIS REVIEW

Because required reporting of pay ratio is as recent as 2018, the volume of research is expected to increase over time. We anticipate updating the review at approximately 5 year intervals based on availability of new research.

## DATA AND ANALYTIC CODE

We will submit supplementary material including data coding sheets and codebook.

## SOURCES OF SUPPORT

**Internal sources**

This research is funded by an HJ Heinz II Chair to the first author.

## Supporting information

Supporting information.

## Data Availability

Data will be made available upon completion of review, including codebook and excel file of study codings.
