# Supplementary material for: PROTOCOL: Is the CEO/employee pay ratio related to firm performance in publicly traded companies?
Source: Campbell Syst Rev. 2024 Nov 4;20(4):e70003. doi: 10.1002/cl2.70003 (PMC11534641; doi:10.1002/cl2.70003)
Supplement: Supplementary file 1 — Supporting information. [file CL2-20-e70003-s001.docx]

# Appendices

## 1 Example - Search strategies:

ABI/INFORM (ProQuest) 1-1-2018 to present

| **#** | **Searches** |
| --- | --- |
| 1 | MAINSUBJECT(“chief executive officer*” OR employee*) |
| 2 | NOFT(ceo OR ceos OR "chief executive officer*" OR "senior leader*" OR "corporate director*" OR executive* OR employee* OR worker*) |
| 3 | [S1] OR [S2] |
| 4 | MAINSUBJECT.EXACT(“wage differential”) |
| 5 | NOFT ((wage* or pay* or compensation* or salary* or salaries) NEAR/3 (gap* or ratio* or inequalit* or disparit* or dispersion* or relative or differential*)) |
| 6 | [S4] OR [S5] |
| 7 | MAINSUBJECT.EXACT("financial performance" OR "corporate profits" OR earnings OR "retained earnings" OR "undistributed profits" OR "earnings per share" OR "return on investment" OR revenue OR "financial ratios" OR "return on assets" OR "return on equity" OR "corporate mergers") |
| 8 | NOFT(firm* OR corporate OR corporation* OR company OR companies OR organi?ational OR financial OR business) NEAR/5 (performance OR profit* OR "stock price" OR value OR sales OR revenue OR "market share" OR innovation OR growth OR "financial health" OR liquidity OR success OR productiv* OR earning*) |
| 9 | NOFT("return on assets" OR ROA OR "return on income" OR ROI OR ROIC OR "return on invested capital" OR "return on capital" OR ROC OR "return on equity" OR ROE OR EBITDA OR "earning* before interest tax depreciation and amortization" OR "stock market returns" OR "equity pricing" OR "profit margin" OR "financial ratio" OR "merger and acquisition" OR "mergers and acquisitions" OR “earning* per share”) |
| 10 | [S7] OR [S8] OR [S9] |
| 11 | [S3] AND [S6] AND [S10] |
| 12 | Limits: Source type - scholarly journals, dissertations & theses, working papers, conference papers & proceedings |
| 13 | Limit: From 1/1/2018 - present |
